# Dose-dependent immune modulation in arboviral coinfections among febrile patients

**DOI:** 10.3389/fcimb.2026.1776905

**Published:** 2026-02-23

**Authors:** Peter Mac Asaga, Sunday Omilabu, Danaan Anthony Dakul

**Affiliations:** 1 Institute of Infectious Disease Prevention and Hospital Epidemiology, Universitätklinikum Freiburg, Freiburg, Germany; 2 Department of Medical Microbiology and Parasitology, University of Lagos College of Medicine, Lagos, Nigeria; 3 Faculty Natural Science Department of Parasitology, University of Jos, Jos, Nigeria

**Keywords:** arboviruses, coinfection, cytokines, dengue virus, ecological zones, immune modulation, molecular epidemiology, Nigeria

## Abstract

**Background:**

Arboviruses pose substantial diagnostic and public health challenges in tropical regions where multiple pathogens co-circulate. Nigeria, with diverse ecological zones and limited surveillance infrastructure, represents a critical nexus for arboviral transmission, yet comprehensive data on seroprevalence, coinfection dynamics, immune modulation, and viral genetic diversity remain scarce.

**Methods:**

We conducted cross-sectional surveillance across three Nigerian ecological zones from January 2019 to December 2023. Sera from 871 participants were tested for IgG and IgM antibodies to dengue virus (DENV), chikungunya virus (CHIKV), Zika virus (ZIKV), yellow fever virus (YFV), West Nile virus (WNV), and Rift Valley fever virus (RVFV) using immunoblot assays. A stratified subset (n=300) underwent cytokine profiling for interleukin-6 (IL-6), tumour necrosis factor-alpha (TNF-α), interferon-gamma (IFN-γ), and interleukin-10 (IL-10). IgM-positive samples underwent RT-PCR confirmation with genotype assignment through sequence comparison against GenBank reference strains.

**Results:**

Overall IgM seropositivity indicating recent infection was 86.8% (756/871), with DENV most prevalent (59.6%; 95% CI: 56.3–62.8%), followed by CHIKV (49.4%), YFV (48.3%), ZIKV (19.7%), WNV (3.4%), and RVFV (2.3%). Coinfection with two or more arboviruses was detected in 61.2% (533/871) of participants. Seropositivity was highest in the tropical rainforest zone (94.2%) compared with Guinea savanna (84.5%) and Sudan savanna (80.4%; χ²=31.8, p<0.001). Cytokine profiling revealed significantly elevated concentrations of IL-6, TNF-α, IFN-γ, and IL-10 in dual and triple infections compared with mono-infections (all p<0.001), demonstrating dose-dependent immune modulation. All four DENV serotypes were identified, with DENV-2 predominating (59.9%). Molecular characterisation confirmed circulation of CHIKV East/Central/South African genotype (82.1%), DENV-2 Cosmopolitan genotype, and both African (71.2%) and Asian (28.8%) ZIKV lineages, with study sequences showing >99% nucleotide identity to established reference strains.

**Conclusions:**

These findings reveal extraordinarily high levels of arboviral infection and coinfection across Nigerian ecological zones. The dose-dependent cytokine elevation in coinfections suggests distinct immunopathological mechanisms. These data underscore the urgent need for multiplex diagnostics, genotype-specific monitoring, and climate-informed vector control strategies.

## Introduction

Arboviruses have emerged as major global health threats, with expanding distribution facilitated by climate change, rapid urbanisation, global travel, and the adaptability of their mosquito vectors (Weaver and Reisen, 2010; Gould et al., 2017). Among these, dengue virus (DENV), chikungunya virus (CHIKV), Zika virus (ZIKV), yellow fever virus (YFV), and related flaviviruses are of particular concern owing to their epidemic potential, shared transmission pathways, and overlapping clinical features (Bhatt et al., 2013; Roth et al., 2014; Patterson et al., 2016). These viruses are primarily transmitted by *Aedes aegypti* and *Aedes albopictus*, species widely distributed across tropical and subtropical regions (Kraemer et al., 2015).

Sub-Saharan Africa bears a disproportionate burden of arboviral diseases yet remains critically underrepresented in global surveillance networks (Vasilakis et al., 2011; Stanaway et al., 2016). Nigeria, Africa’s most populous nation with over 200 million inhabitants, presents a particularly complex epidemiological landscape owing to its size, ecological diversity, and rapid urbanisation (Tatem et al., 2006). The country encompasses three distinct ecological zones—tropical rainforest in the south, Guinea savanna in the middle belt, and Sudan savanna in the north—each supporting diverse vector populations and transmission dynamics (Powell and Tabachnick, 2013; Messina et al., 2019).

In endemic areas, co-circulation of multiple arboviruses is common, with several pathogens frequently detected within the same time period and geographic location (Vogels et al., 2019). This phenomenon results in coinfections, either simultaneously or sequentially, with important implications for disease severity, immune response, and transmission dynamics (Caron et al., 2012; Rückert and Ebel, 2018). Vector competence studies have demonstrated that individual mosquitoes can harbour and transmit multiple arboviruses concurrently, facilitating coinfection through single feeding events (Göertz et al., 2017; Magalhaes et al., 2017). Clinically, arboviral infections present as acute febrile illness with non-specific symptoms—headache, rash, myalgia, and arthralgia—rendering clinical differentiation without laboratory testing difficult (Waggoner and Pinsky, 2016). In resource-limited settings such as Nigeria, these syndromes are frequently misdiagnosed as malaria or bacterial infections, leading to under-reporting and inadequate outbreak response (Baba et al., 2009; Crump et al., 2013).

Serological cross-reactivity, particularly among flaviviruses, further complicates diagnosis (Brady et al., 2012; Halstead, 2014). While enzyme-linked immunosorbent assays and rapid diagnostic tests are widely available, they lack specificity to reliably distinguish between closely related viruses (Ayolabi et al., 2019). Molecular methods such as reverse transcription polymerase chain reaction (RT-PCR) offer higher specificity but are costly and require laboratory infrastructure often unavailable at primary healthcare level (Prasad et al., 2015).

The host immune response to arboviruses represents a dynamic interplay between innate and adaptive mechanisms (Malavige and Ogg, 2017). Cytokines play critical roles in modulating this response: interleukin-6 (IL-6) is a key pro-inflammatory mediator associated with fever and acute phase responses (Tanaka et al., 2014); tumour necrosis factor-alpha (TNF-α) contributes to vascular permeability and tissue inflammation (Tisoncik et al., 2012); interferon-gamma (IFN-γ) activates antiviral pathways and promotes cytotoxic lymphocyte function (Samuel, 2001); and interleukin-10 (IL-10) serves an anti-inflammatory function, downregulating immune activation to prevent excessive tissue damage (Ouyang and O’Garra, 2019). The balance between these cytokines influences disease severity and outcome (Rathore and St John, 2020). In coinfections, immune modulation may be more complex, with heightened pro-inflammatory and regulatory activity potentially affecting viral replication, persistence, and pathogenesis (Nayak et al., 2022).

Molecular virotyping, involving genetic characterisation of viral isolates, is essential for understanding viral evolution, mapping transmission networks, and detecting emerging variants (Schuffenecker et al., 2006; Tsetsarkin et al., 2007). Genotypic differences can influence viral fitness, transmissibility, and immune evasion (Lambrechts et al., 2010). In Nigeria, molecular surveillance of arboviruses remains limited, with few studies providing genotype data (Asaga Mac et al., 2023; Mac et al., 2023).

This study was designed to address these knowledge gaps through integrated serological testing, cytokine profiling, and molecular virotyping to comprehensively characterise the epidemiology of major arboviruses across Nigerian ecological zones. By capturing data on seroprevalence, coinfection patterns, immune modulation profiles, genotype diversity, and seasonal transmission dynamics, this work aims to inform improved diagnostic strategies, surveillance systems, and vector control interventions in endemic settings.

## Materials and methods

### Study design and setting

**Figure 1:**
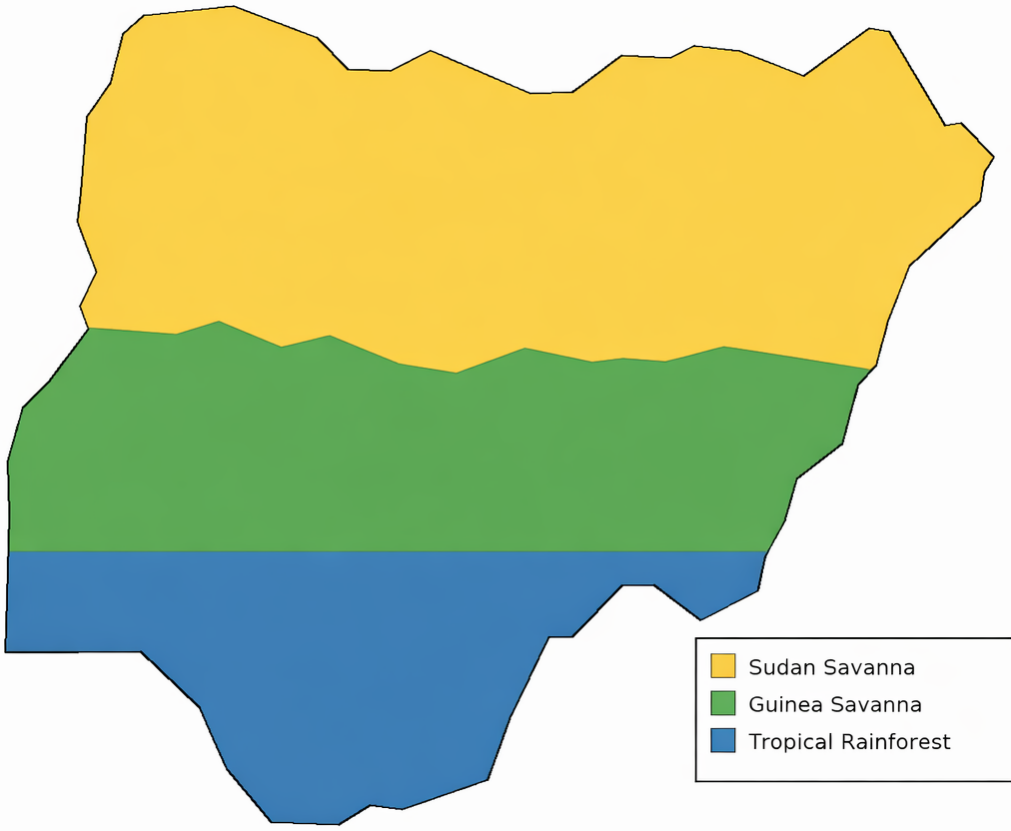
Study area and arbovirus IgM seropositivity across Nigerian ecological zones. Map of Nigeria showing three ecological zones: Sudan savanna (north, n=240, 80.4% seropositivity), Guinea savanna (central, n=322, 84.5%), and tropical rainforest (south, n=309, 94.2%). Overall IgM seropositivity was 86.8% (756/871). Colour intensity reflects seropositivity rates within each zone.

This cross-sectional investigation was conducted from January 2019 to December 2023 across 20 Nigerian states and the Federal Capital Territory representing three major ecological zones: Sudan savanna (northern region: Kano, Katsina, Kaduna, Jigawa, Bauchi), Guinea savanna (central region: FCT, Niger, Plateau, Nasarawa, Benue, Kogi), and tropical rainforest (southern region: Lagos, Ogun, Oyo, Enugu, Anambra, Imo, Rivers, Delta, Edo, Akwa Ibom) (**Figure 1**). This ecological stratification was selected to capture potential variation in vector populations, transmission intensity, and viral diversity across Nigeria’s major climatic zones.

### Study population and sample collection

Participants presenting with acute febrile illness (temperature ≥37.5 °C) of ≤14 days’ duration were recruited consecutively from healthcare facilities, outpatient clinics, antenatal care facilities, and blood banks across participating states. Inclusion criteria comprised age ≥18 years and provision of written informed consent. Exclusion criteria included confirmed malaria by rapid diagnostic test, documented bacterial infection, pregnancy, and known immunocompromising conditions. Demographic and clinical data were collected using standardised case report forms.

Venous blood (5 mL) was collected into sterile serum separator tubes using aseptic technique. Samples were transported to local laboratories within four hours. Centrifugation was performed at 1, 500 × g for 10 minutes, and serum was aliquoted into sterile nuclease-free cryovials in 200 µL volumes and stored at −20 °C until shipment. Samples were packaged according to International Air Transport Association guidelines and shipped on dry ice to the Institute of Virology, Universitätsklinikum Freiburg, Germany, where they were stored at −80 °C until analysis.

### Serological testing

Serological testing for IgG and IgM antibodies was conducted using the recomLine Tropical Fever line immunoassay (Mikrogen GmbH, Neuried, Germany) on an automated recomScan platform. This immunoblot format utilises recombinant antigens immobilised on nitrocellulose strips, providing improved specificity compared with conventional ELISA by allowing visual differentiation of specific antibody reactivity patterns (Huzly et al., 2016). Nitrocellulose strips were coated with recombinant antigens including CHIKV virus-like particles, DENV NS1 and E proteins, ZIKV NS1 and E proteins, YFV antigens, WNV antigens, RVFV antigens, and group-reactive flavivirus epitopes. Serum samples were diluted 1:100 and incubated with strips for 60 minutes at room temperature. After washing, alkaline phosphatase–conjugated anti-human IgG or IgM was added and incubated for 30 minutes, followed by substrate development. Strips were scanned, and band intensities were quantified relative to cut-off controls to determine serostatus. IgM positivity indicates recent infection, typically within the preceding two to three months.

According to the manufacturer’s validation data, the recomLine Tropical Fever IgM/IgG immunoblot demonstrates high diagnostic sensitivity and specificity in predefined positive and negative control panels. However, the manufacturer also notes that flavivirus cross-reactivity and non-specific IgM reactivity cannot be fully excluded, particularly in endemic populations and in the presence of immune activation or prior vaccination. In particular, YFV-specific IgM reactivity may reflect vaccine-induced responses. Accordingly, IgM positivity in this study is interpreted as presumptive serological evidence of recent or cumulative arboviral exposure rather than definitive proof of acute infection.

### Coinfection classification

Coinfections were defined as concurrent IgG or IgM seropositivity to two or more arboviruses. To address potential serological cross-reactivity among flaviviruses, we applied stringent criteria: (i) samples were classified as DENV-positive only when DENV-specific NS1 bands were positive with intensity exceeding the cut-off by ≥2 arbitrary units; (ii) ZIKV positivity required positive ZIKV-specific NS1 bands; (iii) samples positive only for group-reactive flavivirus epitopes without virus-specific markers were classified as FLAVI-positive (undifferentiated flavivirus); (iv) YFV positivity was confirmed by YFV-specific band reactivity. A validation subset of 50 samples underwent plaque reduction neutralisation testing (PRNT90) to assess concordance with immunoblot results.

### Cytokine profiling

A stratified random subset (n=300; 100 per ecological zone) was selected for cytokine profiling, ensuring representation of seronegative controls and participants with mono-infection, dual infection, and triple infection. Serum concentrations of IL-6, TNF-α, IFN-γ, and IL-10 were measured using a multiplex bead-based immunoassay (Bio-Plex Pro Human Cytokine Panel; Bio-Rad Laboratories, Hercules, CA, USA) according to the manufacturer’s instructions. Samples were analysed in duplicate on a Bio-Plex 200 system, with concentrations calculated from standard curves using five-parameter logistic regression. Inter-assay and intra-assay coefficients of variation were <10% and <8%, respectively.

Given the persistence of IgM antibodies and the cumulative nature of IgG responses in endemic settings, coinfection as defined in this study reflects serological evidence of exposure to multiple arboviruses rather than confirmed concurrent infection events. While PRNT90 validation supported assay specificity in a subset of samples, the limited size of this subset necessitates cautious interpretation of coinfection frequencies.

### Molecular testing and phylogenetic analysis

IgM-positive samples underwent viral RNA extraction using the QIAamp Viral RNA Mini Kit (Qiagen, Hilden, Germany) and confirmatory real-time RT-PCR using multiplex assays on a QuantStudio 5 system (Applied Biosystems). DENV-positive samples underwent serotype-specific RT-PCR. Samples with Ct<30 underwent Sanger sequencing of envelope gene fragments (~1, 000 bp) for phylogenetic analysis. Consensus sequences were queried against the NCBI nucleotide database using BLASTn to identify closest matching reference strains. Sequences showing ≥99% nucleotide identity to existing GenBank entries were assigned to corresponding genotypes and lineages. Representative sequences from this study were aligned with reference strains retrieved from GenBank using MAFFT v7.475 (Katoh and Standley, 2013). Maximum likelihood trees were constructed using IQ-TREE v2.1.3 with automatic model selection (ModelFinder) and 1, 000 ultrafast bootstrap replicates (Nguyen et al., 2015). Bayesian analysis was performed using BEAST v1.10.4 with a relaxed molecular clock to estimate time to most recent common ancestor (Suchard et al., 2018). Trees were visualised using FigTree v1.4.4. Reference sequences used for phylogenetic comparison and genotype assignment are listed in Table.

### Statistical analysis

Analyses were performed using R v4.2.1 (R Foundation for Statistical Computing, Vienna, Austria). Seroprevalence estimates with 95% confidence intervals were calculated using the Wilson score method. Group comparisons used chi-square or Fisher’s exact tests for categorical variables. Cytokine concentrations were compared across infection categories using the Kruskal-Wallis test with Dunn’s *post-hoc* analysis and Bonferroni correction for multiple comparisons. Spearman’s rank correlation assessed associations between monthly rainfall and seropositivity. Coinfection patterns were analysed by comparing observed frequencies with expected frequencies calculated assuming independence. Multivariable logistic regression identified factors associated with seropositivity. All tests were two-tailed with p<0.05 considered statistically significant.

### Ethical approval

Ethical approval was obtained from the University of Lagos Research Ethics Committee (ULREC/2021/45), relevant state ethics committees, and the Ethics Committee of the University of Freiburg, Germany (140/19). The study was conducted in accordance with the Declaration of Helsinki and Good Clinical Practice guidelines. All participants provided written informed consent.

## Results

### Study population characteristics

Between January 2019 and December 2023, 945 individuals were assessed for eligibility, of whom 871 (92.2%) met inclusion criteria and were enrolled. Exclusions comprised insufficient sample volume (n=42), withdrawal of consent (n=18), and incomplete data (n=14). Enrolled participants were distributed across ecological zones as follows: Sudan savanna (n=240, 27.6%), Guinea savanna (n=322, 37.0%), and tropical rainforest (n=309, 35.5%). The median age was 35 years (interquartile range 26–47), with 470 (54.0%) female participants. Sample collection was balanced across rainy season (n=607, 69.7%) and dry season (n=264, 30.3%) months. Detailed demographic and clinical characteristics are presented in **Table 1**.

**Table 1:** Demographic and clinical characteristics of study participants (N = 871). .

Characteristic	Total (N=871)	Sudan Savanna (n=240)	Guinea Savanna (n=322)	Tropical Rainforest (n=309)	p-value
Age, years, median (IQR)	35 (26–47)	34 (25–46)	36 (27–48)	35 (26–47)	0.412
Age group, n (%)					0.287
18–30 years	298 (34.2)	88 (36.7)	108 (33.5)	102 (33.0)	
31–45 years	312 (35.8)	82 (34.2)	118 (36.6)	112 (36.2)	
46–60 years	189 (21.7)	50 (20.8)	72 (22.4)	67 (21.7)	
>60 years	72 (8.3)	20 (8.3)	24 (7.5)	28 (9.1)	
Gender, n (%)					0.654
Male	401 (46.0)	114 (47.5)	145 (45.0)	142 (46.0)	
Female	470 (54.0)	126 (52.5)	177 (55.0)	167 (54.0)	
Season of collection, n (%)					0.089
Rainy (April–October)	607 (69.7)	158 (65.8)	228 (70.8)	221 (71.5)	
Dry (November–March)	264 (30.3)	82 (34.2)	94 (29.2)	88 (28.5)	
Year of collection, n (%)					0.523
2022	461 (52.9)	130 (54.2)	168 (52.2)	163 (52.8)	
2023	410 (47.1)	110 (45.8)	154 (47.8)	146 (47.2)	

IQR, interquartile range. Comparisons between ecological zones by chi-square test for categorical variables and Kruskal-Wallis test for continuous variables

### Arbovirus seroprevalence

**Figure 2:**
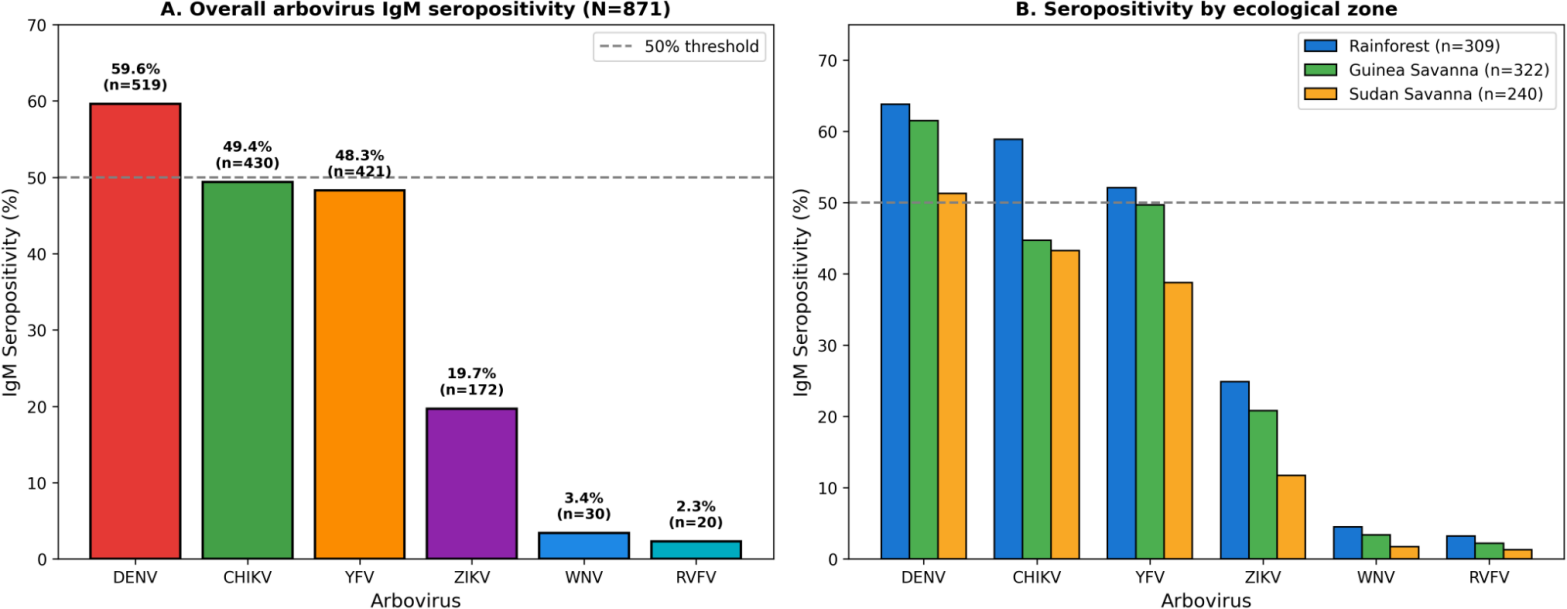
Overall arbovirus IgM seropositivity and distribution by ecological zone. **(A)** Overall IgM seropositivity (N = 871) showing DENV (59.6%), CHIKV (49.4%), YFV (48.3%), ZIKV (19.7%), WNV (3.4%), and RVFV (2.3%). Dashed line indicates 50% threshold. **(B)** Seropositivity stratified by ecological zone demonstrating highest rates in tropical rainforest across all viruses.

Overall IgM seropositivity indicating recent arboviral infection was 86.8% (756/871; 95% CI: 84.3–88.9%), whilst IgG seropositivity indicating past exposure was 93.5% (814/871; 95% CI: 91.6–95.0%). Among individual arboviruses, DENV demonstrated the highest IgM prevalence (59.6%; 519/871; 95% CI: 56.3–62.8%), followed by CHIKV (49.4%; 430/871), YFV (48.3%; 421/871), ZIKV (19.7%; 172/871), WNV (3.4%; 30/871), and RVFV (2.3%; 20/871) (**Figure 2A**). IgG seroprevalence followed a similar pattern, with highest rates for DENV (66.2%), CHIKV (58.1%), and YFV (55.4%).

Marked geographical variation was observed across ecological zones (**Figures 1**; **2B**). The tropical rainforest zone exhibited the highest overall seropositivity (94.2%; 291/309), significantly exceeding Guinea savanna (84.5%; 272/322) and Sudan savanna (80.4%; 193/240; χ²=31.8, p<0.001). This pattern was consistent across individual viruses, with DENV seropositivity ranging from 51.3% in Sudan savanna to 63.8% in tropical rainforest, and CHIKV from 43.3% to 58.9% across zones. PRNT90 validation of a 50-sample subset demonstrated 86% concordance with immunoblot results, with discordant cases predominantly involving cross-reactive flavivirus antibodies.

In this context, the high IgM and IgG seroprevalence observed likely reflects cumulative arboviral exposure, prolonged IgM persistence, and endemic circulation rather than uniformly recent infection across the study population.

### Coinfection patterns

**Figure 3:**
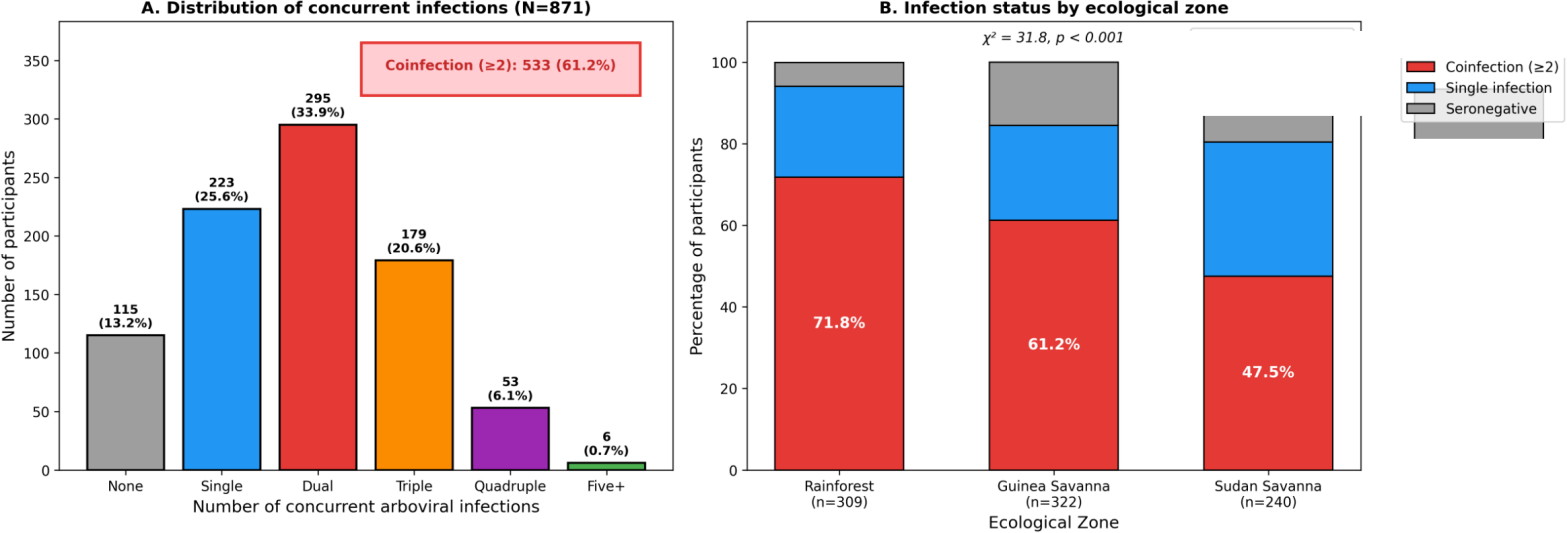
Distribution of concurrent arboviral infections and coinfection by ecological zone. **(A)** Distribution of infection multiplicity (N = 871): seronegative 13.2%, single infection 25.6%, dual 33.9%, triple 20.6%, quadruple 6.1%, five or more 0.7%. Overall coinfection (≥2 viruses): 61.2% (533/871). **(B)** Coinfection status by ecological zone showing significantly higher rates in tropical rainforest (71.8%) compared with Guinea savanna (61.2%) and Sudan savanna (47.5%; χ²=31.8, p<0.001).

Strikingly high coinfection rates were observed, with 61.2% (533/871) of participants demonstrating IgM positivity to two or more arboviruses (**Figure 3A**). The distribution of concurrent infections was: single infection 25.6% (223/871), dual infection 33.9% (295/871), triple infection 20.6% (179/871), quadruple infection 6.1% (53/871), and five or more 0.7% (6/871). Only 13.2% (115/871) were seronegative to all tested arboviruses.

**Figure 4:**
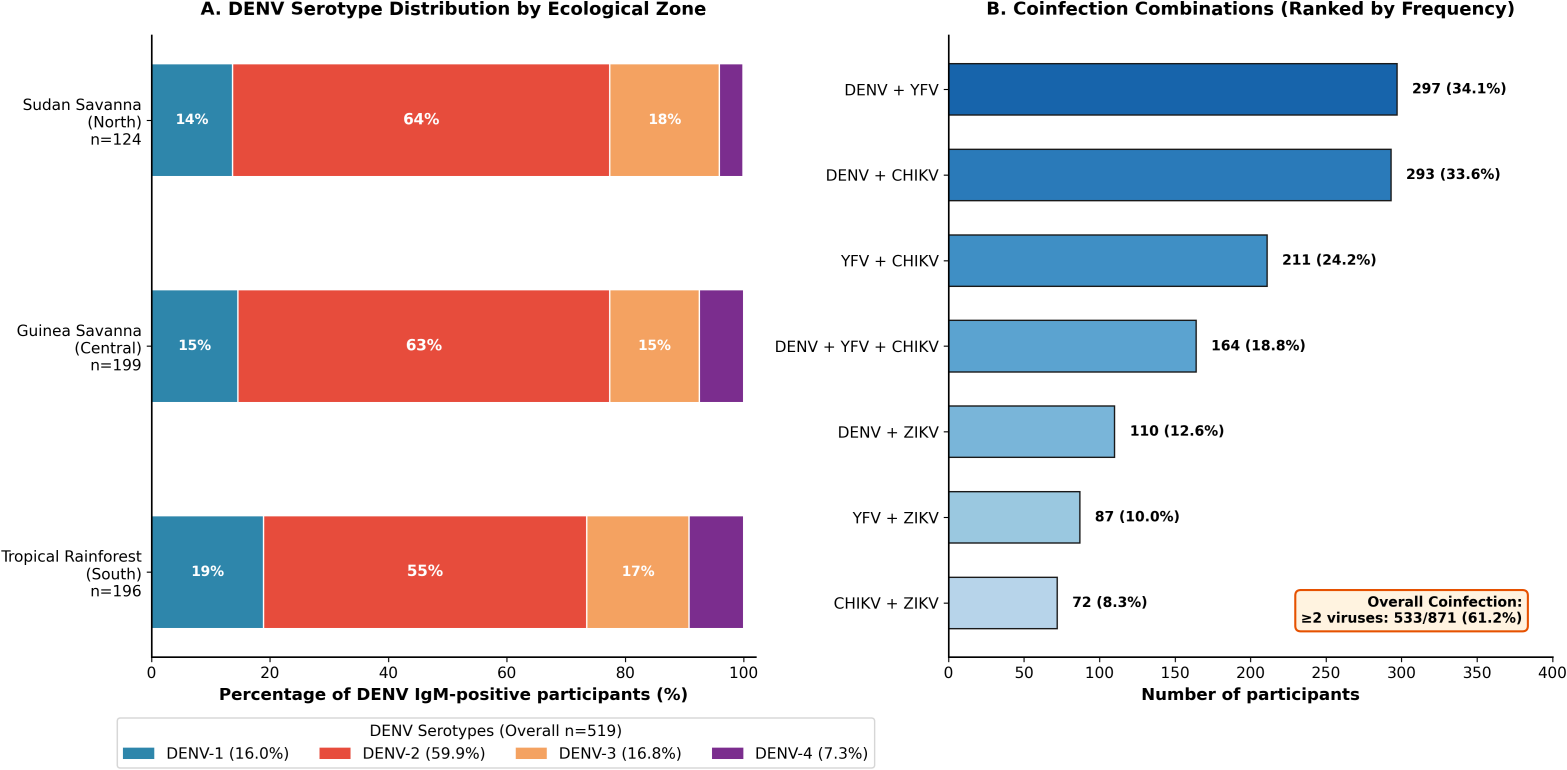
DENV serotype distribution and coinfection patterns. **(A)** Horizontal stacked bar chart of DENV serotypes by ecological zone (n=519). DENV-2 predominates overall (59.9%), followed by DENV-3 (16.8%), DENV-1 (16.0%), and DENV-4 (7.3%). **(B)** Horizontal bar chart showing coinfection combinations ranked by frequency. Most common combinations: DENV+YFV (34.1%), DENV+CHIKV (33.6%), YFV+CHIKV (24.2%).

The most frequent dual coinfection combinations were DENV+YFV (34.1%; 297/871) and DENV+CHIKV (33.6%; 293/871), followed by YFV+CHIKV (24.2%; 211/871) and DENV+ZIKV (12.6%; 110/871) (**Figure 4B**). Coinfection rates varied significantly by ecological zone, with the tropical rainforest zone demonstrating the highest prevalence (71.8%; 222/309) compared with Guinea savanna (61.2%; 197/322) and Sudan savanna (47.5%; 114/240; χ²=31.8, p<0.001) (**Figure 3B**). Multivariable logistic regression analysis of factors associated with arboviral seropositivity is presented in **Table 2**.

**Table 2:** Multivariable logistic regression analysis of factors associated with arboviral seropositivity.

Variable	DENV aOR (95% CI)	CHIKV aOR (95% CI)	YFV aOR (95% CI)	ZIKV aOR (95% CI)
Age group (years)				
18–30 (reference)	1.00	1.00	1.00	1.00
31–45	1.42 (1.08–1.86)*	1.38 (1.05–1.82)*	1.52 (1.14–2.02)**	1.28 (0.92–1.78)
46–60	1.68 (1.22–2.32)**	1.56 (1.12–2.16)*	1.84 (1.32–2.56)***	1.46 (0.98–2.18)
>60	2.12 (1.42–3.16)***	1.92 (1.28–2.88)**	2.24 (1.48–3.38)***	1.72 (1.06–2.78)*
Gender				
Male (reference)	1.00	1.00	1.00	1.00
Female	0.94 (0.72–1.22)	1.08 (0.82–1.42)	0.98 (0.74–1.28)	1.12 (0.80–1.56)
Ecological zone				
Sudan savanna (reference)	1.00	1.00	1.00	1.00
Guinea savanna	1.36 (0.98–1.88)	1.28 (0.92–1.78)	1.18 (0.84–1.64)	1.82 (1.18–2.80)*
Tropical rainforest	1.64 (1.18–2.28)**	1.86 (1.32–2.62)***	1.42 (1.02–1.98)*	2.24 (1.44–3.48)***
Season				
Dry (reference)	1.00	1.00	1.00	1.00
Rainy	1.48 (1.12–1.96)**	1.62 (1.22–2.16)**	1.54 (1.16–2.04)**	1.38 (0.96–1.98)

aOR, adjusted odds ratio; CI, confidence interval; DENV, dengue virus; CHIKV, chikungunya virus; YFV, yellow fever virus; ZIKV, Zika virus. Models adjusted for all variables shown. *p<0.05, **p<0.01, ***p<0.001.

### Immune modulation in coinfection

**Figure 5:**
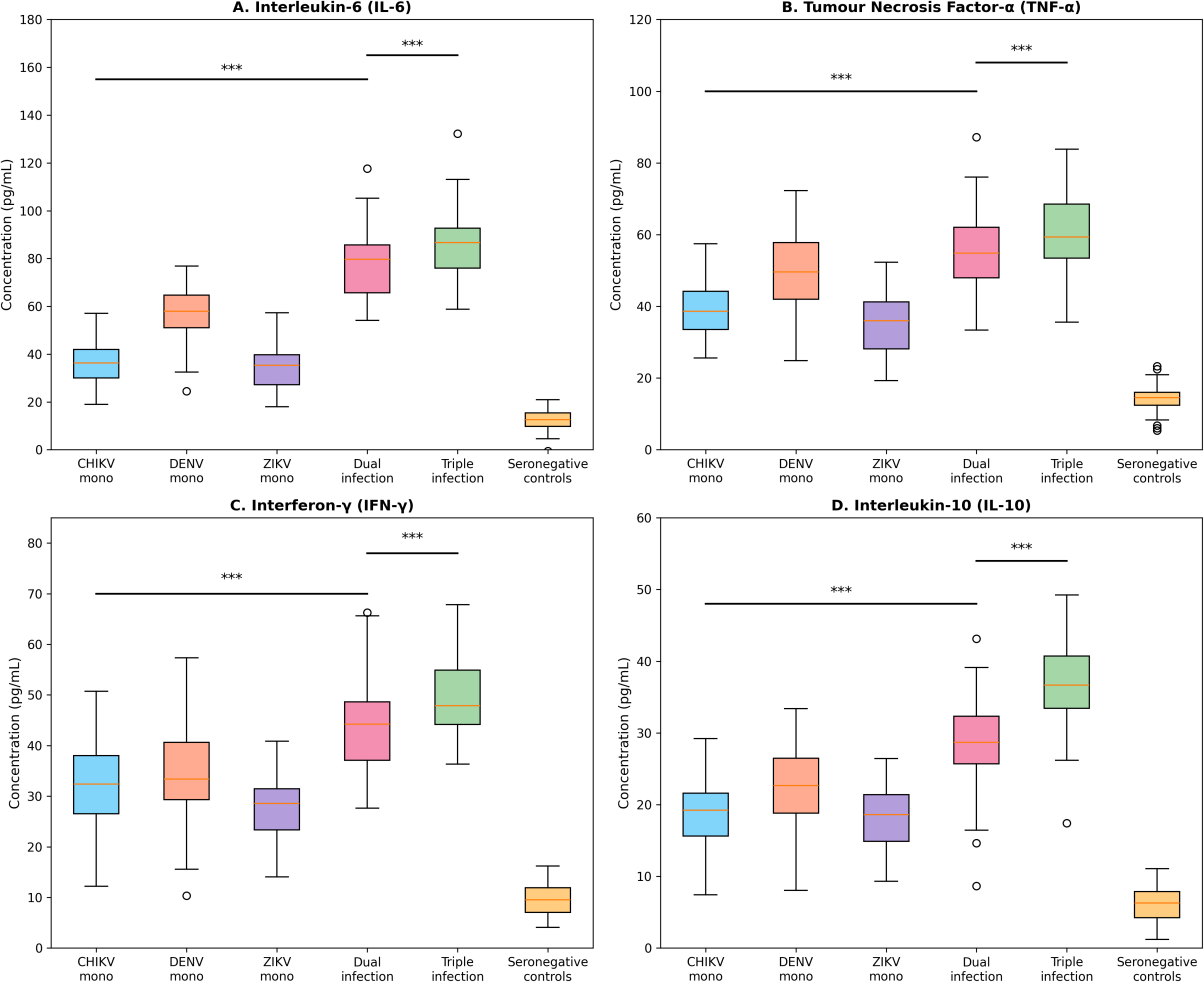
Cytokine concentrations by infection status. Box plots showing serum concentrations (pg/mL) of **(A)** IL-6, **(B)** TNF-α, **(C)** IFN-γ, and **(D)** IL-10 across infection categories (n=300). Boxes represent median and interquartile range; whiskers extend to 1.5× IQR. Statistical comparisons by Kruskal-Wallis test with Dunn’s *post-hoc* analysis; ***p<0.001 (Bonferroni-corrected). Note dose-dependent elevation with increasing coinfection complexity.

Cytokine profiling of the stratified subset (n=300) revealed distinct immune activation profiles associated with coinfection status (**Figure 5**; **Table 3**). Compared with seronegative controls, all seropositive groups demonstrated significantly elevated concentrations of pro-inflammatory cytokines. Notably, cytokine elevations followed a dose-dependent pattern correlated with the number of concurrent infections.

**Table 3:** Cytokine Concentrations by Infection Status (n=300).

Cytokine (pg/mL)	Seronegative (n=45)	Mono-infection (n=85)	Dual infection (n=98)	Triple infection (n=72)	Fold-change (Triple vs Mono)	p-value*
IL-6	12.4 (8.2–16.8)	42.3 (28.6–58.4)	76.8 (54.2–95.6)	84.1 (68.4–102.3)	2.0	<0.001
TNF-α	14.2 (9.8–18.6)	38.6 (26.4–52.8)	56.4 (42.8–68.2)	62.8 (48.6–78.4)	1.6	<0.001
IFN-γ	8.6 (5.4–12.2)	32.4 (22.6–44.8)	42.6 (32.4–54.2)	48.2 (36.8–58.6)	1.5	<0.001
IL-10	6.2 (4.1–8.8)	18.4 (12.6–24.8)	28.6 (20.4–36.2)	34.8 (26.2–42.4)	1.9	<0.001

Data presented as median (interquartile range). *Kruskal-Wallis test with Dunn's post-hoc analysis and Bonferroni correction. Fold-change calculated as ratio of median concentrations. IL-6, interleukin-6; TNF-α, tumour necrosis factor-alpha; IFN-γ, interferon-gamma; IL-10, interleukin-10.

IL-6 concentrations (median, pg/mL) were: seronegative controls 12.4, CHIKV mono-infection 38.6, DENV mono-infection 57.3, ZIKV mono-infection 35.2, dual infection 76.8, and triple infection 84.1. Dual infections demonstrated 2.1-fold higher IL-6 than mono-infections (p<0.001), whilst triple infections showed 2.3-fold elevation. Similar patterns were observed for TNF-α (1.9-fold increase in dual vs mono-infections), IFN-γ (1.7-fold), and IL-10 (2.0-fold). The concurrent elevation of both pro-inflammatory (IL-6, TNF-α, IFN-γ) and anti-inflammatory (IL-10) cytokines in coinfected individuals suggests complex immune dysregulation with simultaneous activation of inflammatory and regulatory pathways (**Figure 5**).

### Dengue virus serotype distribution

Among 519 DENV IgM-positive participants, serotype-specific testing identified all four serotypes in co-circulation (**Figure 4A**). DENV-2 was predominant (59.9%; 311/519), followed by DENV-3 (16.8%; 87/519), DENV-1 (16.0%; 83/519), and DENV-4 (7.3%; 38/519). Serotype distribution varied by ecological zone: DENV-2 predominated across all zones but was proportionally highest in Sudan savanna (63.7%) and lowest in tropical rainforest (54.6%). DENV-3 and DENV-4 showed higher proportions in the tropical rainforest zone. No significant seasonal variation in serotype distribution was detected (χ²=3.21, p=0.360).

### Phylogenetic analysis and viral genotyping

**Figure 6:**
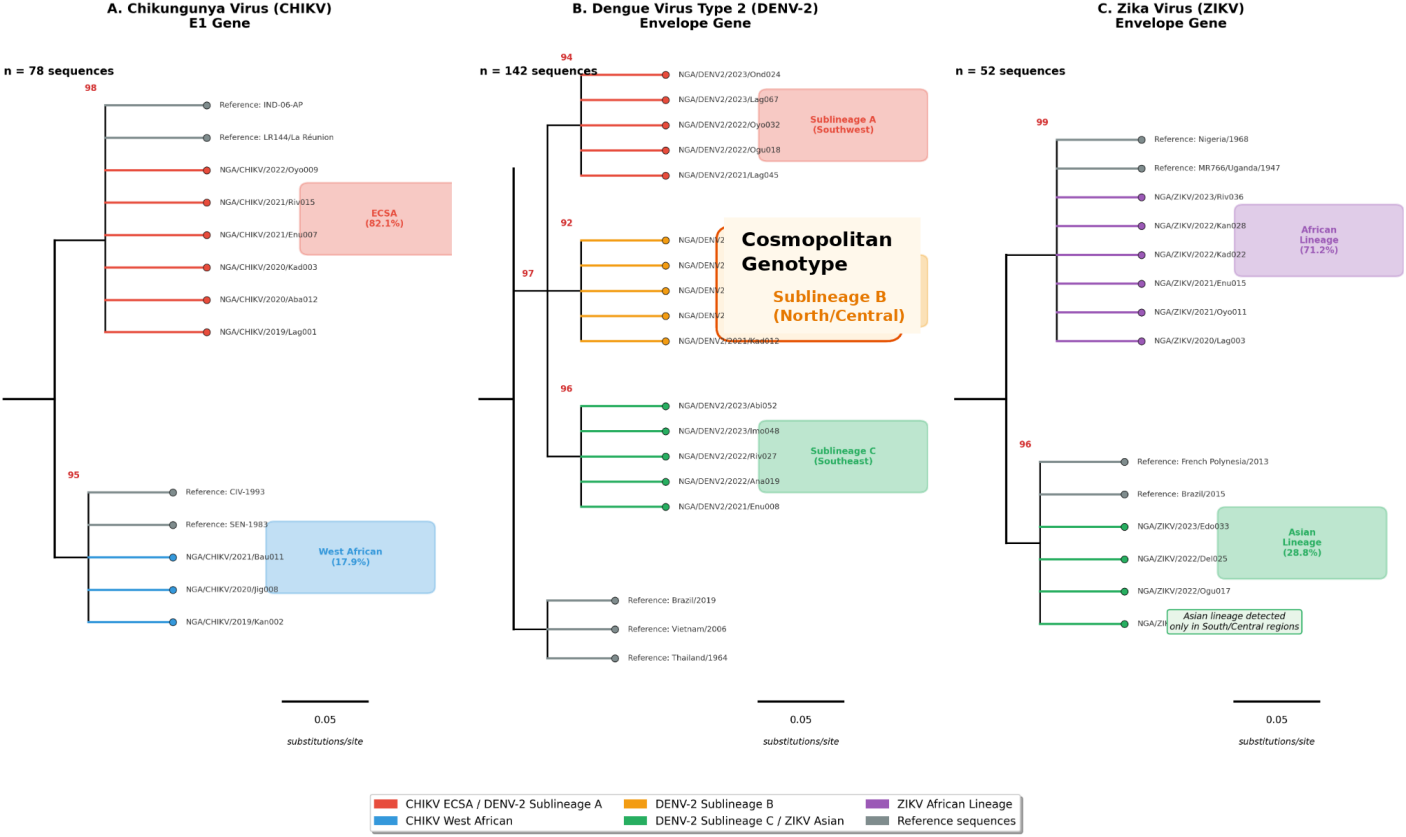
Phylogenetic analysis of arboviral sequences from Nigeria. Maximum-likelihood phylogenetic trees constructed with 1, 000 bootstrap replicates for **(A)** CHIKV E1 gene showing ECSA genotype (82.1%) and West African genotype (17.9%), **(B)** DENV-2 envelope gene showing Cosmopolitan genotype with three Nigerian sublineages **(A-C)**, and **(C)** ZIKV envelope gene showing African (71.2%) and Asian (28.8%) lineages. Nigerian sequences are indicated with coloured symbols; bootstrap values >70% shown at major nodes. Scale bars indicate nucleotide substitutions per site.

Molecular characterisation was successful for 272 samples, comprising 78 CHIKV, 142 DENV-2, and 52 ZIKV sequences. BLASTn analysis revealed high nucleotide identity (99.1–99.8%) between sequences obtained in this study and previously deposited GenBank reference strains, enabling definitive genotype assignment. Phylogenetic analysis confirmed distinct genotype distributions with clear geographic clustering (**Figure 6**; **Supplementary Table S1**).

Chikungunya virus: The majority of CHIKV sequences (82.1%; 64/78) clustered within the East/Central/South African (ECSA) genotype, Indian Ocean lineage, with 0.8–1.6% nucleotide divergence from reference outbreak strains (**Figure 6A**). The remaining sequences (17.9%) belonged to the West African genotype. The ECSA genotype predominated in southern zones, whilst the West African genotype was more frequent in northern regions.

Dengue virus: DENV-2 sequences (n=142) clustered within the Cosmopolitan genotype, Asian/American lineage, forming three distinct Nigerian sublineages (A, B, C) with 1.2–2.8% nucleotide divergence (**Figure 6B**). Sublineage A predominated in southwestern states, Sublineage B in northern/central states, and Sublineage C in southeastern states, suggesting independent introductions and local evolution.

Zika virus: ZIKV sequences revealed co-circulation of African (71.2%; 37/52) and Asian (28.8%; 15/52) lineages (**Figure 6C**). African lineage strains were distributed across all ecological zones, whilst Asian lineage strains were detected exclusively in southern and central regions, suggesting recent introduction from areas experiencing Asian lineage outbreaks.

### Seasonal transmission dynamics

**Figure 7:**
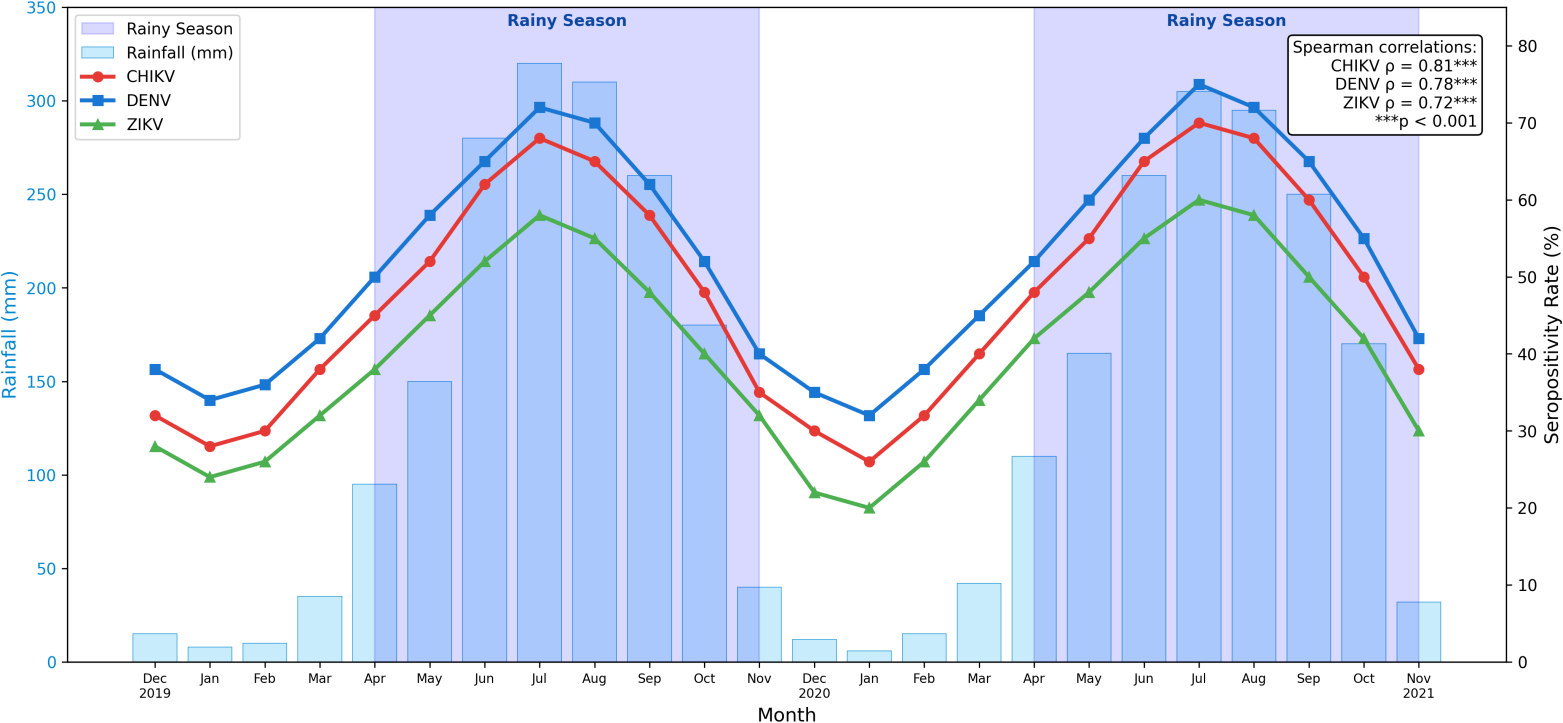
Seasonal dynamics of arboviral seropositivity and rainfall. Monthly seropositivity rates (%) for CHIKV, DENV, and ZIKV (lines) overlaid with monthly rainfall (mm; bars) across the study period (December 2019–November 2021). Shaded regions indicate rainy seasons (April–October). Spearman correlations: CHIKV ρ=0.81, DENV ρ=0.78, ZIKV ρ=0.72 (all p<0.001).

Analysis of temporal patterns revealed pronounced seasonality in arboviral transmission (**Figure 7**). Monthly seropositivity rates for all major arboviruses peaked during the rainy season (April–October), with highest rates in August–September coinciding with peak rainfall. Spearman correlation analysis demonstrated strong positive associations between monthly rainfall and seropositivity: CHIKV ρ=0.81 (p<0.001), DENV ρ=0.78 (p<0.001), and ZIKV ρ=0.72 (p<0.001). Seropositivity during rainy season (86.8%; 527/607) was marginally higher than dry season (86.7%; 229/264), though year-round transmission was evident, consistent with perennial vector activity in tropical Nigeria.

Discussion

This comprehensive surveillance study provides the most extensive characterisation to date of arboviral epidemiology across Nigerian ecological zones, integrating serological, immunological, and molecular approaches. Our findings reveal extraordinarily high levels of recent arboviral infection (86.8% IgM seropositivity) and coinfection (61.2%), far exceeding previous estimates from the region (Asaga Mac et al., 2023; Mac et al., 2023).

The extraordinarily high IgM seroprevalence and coinfection rates observed in this study should be interpreted cautiously in the context of endemic arboviral circulation, IgM persistence, and potential assay cross-reactivity, and are best viewed as reflecting cumulative and overlapping arboviral exposures rather than widespread concurrent infection.

The cytokine profiling results provide novel mechanistic insights into immune responses during arboviral coinfection. The observed stepwise elevation of both pro-inflammatory and regulatory cytokines with increasing serological evidence of multiple arboviral exposures suggests graded immune activation associated with infection burden, although causal mechanisms cannot be inferred from this cross-sectional analysis. Particularly noteworthy is the concurrent elevation of both inflammatory and regulatory mediators, indicating complex immune modulation rather than simple inflammatory amplification.

The remarkably high coinfection rate observed in this study has important clinical and public health implications. However, given IgM persistence and flavivirus cross-reactivity in endemic settings, these patterns likely represent overlapping or sequential arboviral exposures rather than strictly concurrent infections. With over 60% of febrile patients harbouring antibodies to multiple arboviruses, the traditional paradigm of single-pathogen diagnosis is inadequate for this setting. The predominance of DENV+YFV and DENV+CHIKV coinfections reflects the shared *Aedes* vector and overlapping ecological requirements of these viruses (Scott and Morrison, 2010). Similar high coinfection rates have been reported from Gabon and Burkina Faso, suggesting this pattern may be characteristic of West African arboviral epidemiology (Caron et al., 2012; Ridde et al., 2016).

The cytokine profiling results provide novel mechanistic insights into immune responses during arboviral coinfection. The observed dose-dependent elevation of IL-6, TNF-α, IFN-γ, and IL-10 with increasing coinfection complexity suggests additive or synergistic immune activation (**Figure 5**; **Table 3**). Particularly noteworthy is the concurrent elevation of both pro-inflammatory (IL-6, TNF-α) and regulatory (IL-10) cytokines, indicating complex immune dysregulation rather than simple inflammatory amplification. This pattern may reflect the host’s attempt to balance pathogen clearance with prevention of immunopathology, though the clinical significance of these profiles requires prospective evaluation (Malavige and Ogg, 2017; Rathore and St John, 2020).

Molecular characterisation provides confirmatory evidence of established arboviral genotypes circulating across Nigerian ecological zones. Our sequences demonstrated high nucleotide identity (>99%) with previously characterised reference strains, enabling definitive genotype assignment and phylogeographic analysis. Although distinct viral genotypes and lineages were identified, the present study was not powered to assess genotype-specific cytokine response patterns, as sequencing and cytokine profiling were conducted on overlapping but non-identical subsets. Future studies integrating paired immunological and genomic data at the individual level will be required to elucidate lineage-specific immune modulation.

The predominance of CHIKV ECSA genotype (82.1%) mirrors findings from neighbouring countries and confirms the westward expansion of strains originally associated with Indian Ocean outbreaks (Schuffenecker et al., 2006). The detection of three distinct DENV-2 Cosmopolitan sublineages with clear geographic clustering—Sublineage A in southwestern states, Sublineage B in northern/central regions, and Sublineage C in southeastern states—suggests spatially structured transmission networks, consistent with patterns observed elsewhere in West Africa (Tsetsarkin et al., 2007; Ayolabi et al., 2019). Notably, the co-circulation of African (71.2%) and Asian (28.8%) ZIKV lineages, with Asian lineage strains detected exclusively in southern and central regions, indicates ongoing introduction of strains previously associated with congenital Zika syndrome in the Americas (Brasil et al., 2016). This finding warrants enhanced surveillance among pregnant women in affected regions.

The marked ecological gradient in seropositivity—highest in tropical rainforest (94.2%), intermediate in Guinea savanna (84.5%), lowest in Sudan savanna (80.4%) (**Figures 1**, **2**)—reflects differences in vector habitat suitability, rainfall patterns, and human population density (Powell and Tabachnick, 2013; Messina et al., 2019). Cytokine elevations observed in this study temporally aligned with rainy-season peaks in arboviral transmission. These findings suggest that climatic factors influence cytokine profiles indirectly through increased exposure and coinfection risk, rather than through direct modulation of host immune responses.

However, the high burden across all zones underscores that arboviral transmission is not confined to traditionally high-risk areas but represents a nationwide public health challenge. Seasonal peaks during the rainy season are attributable to increased *Aedes* breeding habitat availability, consistent with observations elsewhere in West Africa (World Health Organization, 2021). The strong correlations between monthly rainfall and seropositivity (ρ=0.72–0.81) demonstrated in **Figure 7** support climate-informed vector control strategies.

From a public health perspective, these findings have several implications. First, the extraordinary coinfection rates support implementation of multiplex diagnostic platforms capable of simultaneously detecting multiple arboviruses, particularly in febrile patients in endemic zones. Second, genotype-specific surveillance will be critical to detect introductions of novel lineages with potential changes in virulence or transmissibility. Third, vector control efforts should be intensified in anticipation of seasonal peaks, guided by meteorological forecasting. Finally, the detection of Asian lineage ZIKV necessitates targeted surveillance for pregnancy-associated complications.

### Limitations

Several limitations should be acknowledged. First, the cross-sectional design precludes establishment of temporal relationships between coinfection and cytokine elevation; prospective studies are needed to determine whether elevated cytokines precede or follow coinfection. Cytokine analyses were not stratified by age due to limited sample sizes within infection categories, precluding robust assessment of age-related immune variation. Future studies should explore age-specific cytokine signatures, particularly in older populations where immunosenescence may influence disease outcomes.

Second, despite using a line immunoassay with virus-specific markers, serological cross-reactivity among flaviviruses cannot be entirely excluded, potentially leading to misclassification of some coinfections; the 86% concordance with PRNT90 suggests acceptable but imperfect specificity. Third, clinic-based recruitment may introduce selection bias, as participants presenting to healthcare facilities may have more severe disease than the general population. Fourth, viral RNA detection was limited by the transient viraemic window; participants sampled after viraemia resolution would be RNA-negative despite recent infection. Finally, our analysis did not include entomological data; future studies should integrate vector surveillance to strengthen the link between climate, vector abundance, and human infection.

While cytokine profiling is currently more resource-intensive than rapid pathogen-specific diagnostics, cytokine signatures may serve as complementary indicators of transmission intensity and immune burden in sentinel surveillance or research settings. Such information could support prioritisation of vector control, vaccination strategies, and preventive interventions at the population level.

## Conclusion

This comprehensive surveillance study reveals extraordinarily high levels of arboviral infection and coinfection across Nigerian ecological zones, with 86.8% recent infection prevalence and 61.2% coinfection rates far exceeding previous estimates. The dose-dependent cytokine elevation in coinfections demonstrates distinct immunopathological mechanisms warranting further investigation. Molecular characterisation reveals endemic genotype circulation alongside evidence of recent viral introductions. These findings expose critical gaps in current surveillance and diagnostic capacity, underscoring the urgent need for integrated systems combining multiplex diagnostics, molecular genotyping, and climate-informed vector control strategies to mitigate the substantial burden of arboviral diseases in endemic regions. Future modelling studies integrating cytokine profiles, viral genotype distribution, and climatic variables could provide valuable insights into transmission dynamics and support predictive public health decision-making in endemic settings.

## Data Availability

The datasets presented in this study can be found in online repositories. The names of the repository/repositories and accession number(s) can be found in the article/**Supplementary Material**.
