# Supplementary material for: Dose-dependent immune modulation in arboviral coinfections among febrile patients
Source: Front Cell Infect Microbiol. 2026 Feb 23;16:1776905. doi: 10.3389/fcimb.2026.1776905 (PMC12968230; doi:10.3389/fcimb.2026.1776905)
Supplement: Supplementary file 1 [file Table1.docx]

**Supplementary Table S1. Reference Sequences Used for Phylogenetic Analysis and Genotype Assignment**

| **Virus/Genotype** | **Reference Strain** | **GenBank Accession** | **Origin** | **Identity (%)** |
| --- | --- | --- | --- | --- |
| ***CHIKV — ECSA genotype*** |  |  |  |  |
|  | LR2006-OPY1 | DQ443544 | La Réunion, 2006 | 99.2–99.6 |
|  | IND-06-AP3 | EF027134 | India, 2006 | 99.1–99.5 |
|  | Tanzania-1953 | AF369024 | Tanzania, 1953 | 97.8–98.2 |
| ***CHIKV — West African genotype*** |  |  |  |  |
|  | SEN-1983 | HM045823 | Senegal, 1983 | 99.4–99.7 |
|  | CIV-1993 | HM045817 | Côte d'Ivoire, 1993 | 99.2–99.6 |
|  | NIG-1964 | HM045819 | Nigeria, 1964 | 98.6–99.1 |
| ***DENV-2 — Cosmopolitan genotype*** |  |  |  |  |
|  | Thailand-1964 | AY158328 | Thailand, 1964 | 98.4–98.8 |
|  | Vietnam-2006 | FJ882570 | Vietnam, 2006 | 99.1–99.4 |
|  | Brazil-2019 | MT415101 | Brazil, 2019 | 99.3–99.6 |
|  | Burkina Faso-2016 | MF004387 | Burkina Faso, 2016 | 99.5–99.8 |
| ***ZIKV — African lineage*** |  |  |  |  |
|  | MR766-Uganda-1947 | AY632535 | Uganda, 1947 | 98.2–98.6 |
|  | Nigeria-1968 | HQ234499 | Nigeria, 1968 | 99.4–99.7 |
|  | Senegal-1984 | KF383117 | Senegal, 1984 | 99.1–99.5 |
| ***ZIKV — Asian lineage*** |  |  |  |  |
|  | French Polynesia-2013 | KJ776791 | French Polynesia, 2013 | 99.2–99.5 |
|  | Brazil-2015 | KU321639 | Brazil, 2015 | 99.3–99.6 |
|  | Puerto Rico-2015 | KU501215 | Puerto Rico, 2015 | 99.1–99.4 |

*Reference sequences were retrieved from GenBank for phylogenetic comparison with sequences obtained from 272 RT-PCR positive samples in this study (CHIKV, n=78; DENV-2, n=142; ZIKV, n=52). Identity percentages represent the range of nucleotide similarity between study isolates and each reference strain as determined by BLASTn analysis. CHIKV, chikungunya virus; DENV-2, dengue virus type 2; ZIKV, Zika virus; ECSA, East/Central/South African genotype.*
